# Medication adherence to lipid-lowering agents after percutaneous coronary intervention: nationwide real-world data in the Netherlands

**DOI:** 10.1007/s12471-026-02028-8

**Published:** 2026-03-02

**Authors:** Marijke J. C. Timmermans, M. Patrick Witvliet, Judith A. van Erkelens, Jan Reitsma, Pieter W. Kamphuisen, Cyril Camaro, Peter W. Danse, E. Karin Arkenbout

**Affiliations:** 1 Netherlands Heart Registration, Utrecht, The Netherlands; 2 Department of Cardiology, Tergooi Medical Centre, Hilversum, The Netherlands; 3 Vektis, Zeist, The Netherlands; 4 Zorgverzekeraars Nederland, Zeist, The Netherlands; 5 Department of Vascular Medicine, Amsterdam University Medical Centre, Amsterdam, The Netherlands; 6 Department of Internal Medicine, Tergooi Medical Centre, Hilversum, The Netherlands; 7 Department of Cardiology, Radboud University Medical Centre, Nijmegen, The Netherlands; 8 Department of Cardiology, Rijnstate Hospital, Arnhem, The Netherlands

**Keywords:** Lipid-lowering medication, Medication adherence, Claims data, Percutaneous Coronary Intervention

## Abstract

**Background:**

Lipid-lowering medication reduces the risk of future cardiovascular events and mortality, yet adherence is often disappointing. This study evaluates adherence rates of lipid-lowering medication and its subtypes during the first year following acute and elective percutaneous coronary intervention (PCI) in the Netherlands.

**Methods:**

This retrospective cohort study utilized data from a nationwide all-payer claims database managed by Vektis, containing all medical care claims reimbursed by Dutch national insurance companies. We included 97,176 patients who underwent PCI in 2018–2020. Adherence was defined as a medication possession rate ≥ 80%.

**Results:**

Adherence rates 0–3 months post-elective PCI ranged from 71–73% among the years and remained stable over the year following PCI. For acute PCI, adherence rates 0–3 months post-acute PCI were initially higher (79–81%) but declined to 74–76% during the year following PCI.

During the year following PCI, adherence rates for ezetimibe and proprotein convertase subtilisin/kexin type 9 (PCSK9) inhibitors increased slightly to about 13%, respectively 2%, while statin adherence decreased. For statin subtypes, adherence rates for rosuvastatin increased at the expense of simvastatin, with adherence for atorvastatin and other statins remaining relatively stable. Lower adherence rates were observed among females and patients ≥ 80 years compared to males and younger patients.

**Conclusion:**

This study found lipid-lowering medication adherence 1 year post-elective PCI ranged from 71–73% and post-acute PCI from 74–76%. Lower adherence rates were observed in women and elderly patients. Adherence rates of ezetimibe and PCSK9 inhibitors increased throughout the year following PCI, while statin use decreased.

**Supplementary Information:**

The online version of this article (10.1007/s12471-026-02028-8) contains supplementary material, which is available to authorized users.

## Introduction

Patients with a history of coronary revascularisation, including acute and elective percutaneous coronary intervention (PCI), are at high risk of future cardiovascular disease (CVD) events. Consequently, secondary preventive treatment with lipid-lowering medication is indicated for all such patients, reducing the risk of future cardiovascular events and mortality by lowering low-density lipoprotein cholesterol (LDL-c) [1–6].

**Fig. 1:**
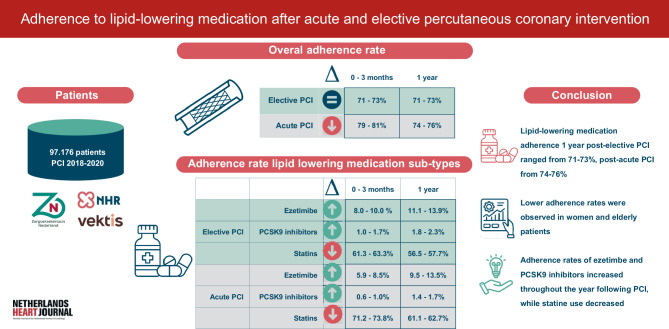
Infographic

Despite the proven benefits of lipid-lowering medication on future CVD events in patients with established CVD, adherence to lipid-lowering medication is often suboptimal [7–14]. These studies primarily focus on statin adherence post-acute myocardial infarction, but there is limited data on adherence rates for various lipid-lowering medication subtypes (statins, ezetimibe and proprotein convertase subtilisin/kexin type 9 (PCSK9) inhibitors), following acute and elective PCI. Therefore, this nationwide study aimed to evaluate adherence rates of lipid-lowering medication with real-world data, specified for statins, ezetimibe, and PCSK9 inhibitors, for one year following acute and elective PCI in the Netherlands (Fig. 1).

## Methods

This study is part of a quality improvement project, conducted by the PCI registration committee of the Netherlands Heart Registration (NHR), in close collaboration with the Dutch Association of Health Insurers (in Dutch: Zorgverzekeraars Nederland) and the Dutch Health Care Information Centre (Vektis). Within the NHR, cardiologists and cardiothoracic surgeons register baseline, procedural, and outcome data for all invasive cardiac interventional, electrophysiological, and surgical procedures. Through public reporting, the NHR provides transparency of outcome data to cardiac patients, healthcare providers, and policymakers [15]. The interpretation of the registered data is primarily managed by registration committees, where designated cardiologists and cardiothoracic surgeons represent their hospitals. When relevant variations in outcomes are observed, the committees discuss healthcare delivery processes and share best practices. Additionally, these registration committees conduct quality improvement projects, including this study.

### Data source

This retrospective observational cohort study utilized data from a nationwide all-payer claims database managed by Vektis, containing medical care claims reimbursed by Dutch national insurance companies. Since 99.9% of the Dutch population has health insurance [16], this database almost covers all medical care provided to the Dutch population. This study obtained claims data on lipid-lowering medication prescriptions for patients undergoing either elective PCI (health care activities 33231-33234) or acute PCI (health care activity 33238) from the Vektis database.

### Study population

This study included all patients undergoing acute or elective PCI from January 1, 2018, to December 31, 2020, in the Netherlands. Patients who died within 365 days post-PCI were excluded, and in cases of multiple PCIs within a calendar year, only the first PCI was included.

### Lipid-lowering treatment and adherence

We utilized the Vektis registry to extract data on whether patients were dispensed lipid-lowering medication at the pharmacy, using Anatomical Therapeutic Chemical (ATC) codes, and to ascertain the duration of the active lipid-lowering medication prescription. We extracted data on whether patients had been prescribed statins (ATC code C10AA01, C10AA03, C10AA04, C10AA05, and C10AA07), ezetimibe (ATC code C10AX09), and/or PCSK9 inhibitor (ATC code C10AX13 or C10AX14) dispensed at the pharmacy 90-1 days prior to PCI, and 1–365days after PCI.

Adherence to lipid-lowering medication was assessed at baseline until one-year post-PCI, defined as a medication possession rate (MPR) ≥ 80% during a specified period. Patients with an active lipid-lowering medication prescription and an MPR below 80% were considered non-adherent. Patients without an active prescription (MPR = 0%) were defined as non-users. A cut-off value of 80% was chosen as this is in line with existing literature [17], and because we aimed to include those patients who used the lipid-lowering medication with a reasonable dosage, while avoiding that patients are wrongly excluded because of administrative mistakes. Patients adherent to lipid-lowering medication were divided into three groups: those prescribed statins, ezetimibe, or PCSK9 inhibitors. We used the most recent prescription in each period to categorize patients. If multiple categories of lipid-lowering medication were dispensed on the same day, categorization followed this order: (1) PCSK9 inhibitors, (2) ezetimibe, and (3) statins. Additionally, we assessed the distribution of adherence rates among different statins subtypes and described trends in prescription behaviour from 2018–2020.

### Statistical methods

Discrete variables were presented as numbers and percentages, and normally distributed continuous variables as mean ± standard deviation (SD). Baseline adherence was evaluated during the three months prior to PCI. For patients who initiated lipid-lowering medication during this period, MPR was calculated from the first dispense date until the PCI date. Adherence rates for lipid-lowering medication were then assessed over the year following PCI, divided into four three-month periods (0–3, 4–6, 7–9, and 10–12 months post-PCI), and reported separately for 2018, 2019, and 2020 to monitor trends. The period 0–3 months post-PCI was referred to as initial adherence, and 10–12 months as long-term adherence. Adherence rates for lipid-lowering medication subtypes (statins, ezetimibe, and PCSK9 inhibitors) and statin subtypes (simvastatin, atorvastatin, rosuvastatin, and others) were also specified. Secondary analyses stratified lipid-lowering medication adherence rates by sex and age to examine differences in sex and adherence rates between patients aged 80 years and older versus patients younger than 80 years. We also performed subgroup analyses for the patients with single PCI and the patients with a recurrent PCI within 1 year. A sensitivity analysis was performed for the patients who died during follow-up.

Additionally, long-term adherence rates for lipid-lowering medication were reported for individual PCI centres to assess clinical practice variations. To mitigate yearly variation effects, data from 2018, 2019, and 2020 were combined for both elective and acute PCI cases. We also classified the PCI centres into regions (i.e., Northern, Eastern, Western, and Southern Netherlands) and into SES-WOA score regions (i.e., score < 0.0 and ≥ 0.0). SES-WOA concern the socioeconomic scores of private households, which are calculated by Statistics Netherlands (CBS) and based on household data concerning welfare (a combination of income and wealth), highest level of education and recent labour participation. To classify the hospitals into categories, we used the geographical map of the Dutch Ministry of Health, Welfare and Sport [18].

All statistics were computed using SAS software (version 9.4, SAS Institute Inc., Cary, NC, USA).

## Results

Between 2018–2020 102,963 patients underwent PCI. We excluded 5787 patients who died within 365 days after PCI. Among the 97,176 included patients, 46,474 (48%) underwent elective PCI and 50,702 (52%) acute PCI. Baseline characteristics are shown in Tab. 1.

**Table 1:** Baseline characteristics study population

	Elective PCI (*n* = 46,474)	Acute PCI (*n* = 50,702)
*Male, n (%)*	33,337 (71.7)	36,576 (72.1)
*Age*		
< 50 years, *n* (%)	1980 (4.3%)	4820 (9.5%)
50 t/m 59 years, *n* (%)	7663 (16.5%)	11,699 (23.1%)
60 t/m 69 years, *n* (%)	13,894 (29.9%)	14,254 (28.1%)
70 t/m 79 years, *n* (%)	15,601 (33.6%)	13,187 (26.0%)
≥ 80 years, *n* (%)	7336 (15.8%)	6742 (13.3%)
*Year of PCI*		
2018, *n* (%)	16,686 (35.9)	16,322 (32.2)
2019, *n* (%)	16,181 (34.8)	17,196 (33.9)
2020, *n* (%)	13,607 (29.3)	17,184 (33.9)
Baseline adherence* (year *=* *2018)*		
Adherent to LLM, %	8577 (51.4%)	4129 (25.3%)
Not adherent to LLM, %	1318 (7.9%)	473 (2.9%)
LLM non-users, %	6791 (40.7%)	11,719 (71.8%)
Baseline adherence* (year *=* *2019)*		
Adherent to LLM, %	9482 (58.6%)	4884 (28.4%)
Not adherent to LLM, %	1618 (10.0%)	619 (3.6%)
LLM non-users, %	5080 (31.4%)	11,693 (68.0%)
Baseline adherence* (year *=* *2020)*		
Adherent to LLM, %	8042 (59.1%)	4966 (28.9%)
Not adherent to LLM, %	1252 (9.2%)	550 (3.2%)
LLM non-users, %	4313 (31.7%)	11,668 (67.9%)

*PCI* percutaneous coronary intervention, *n* number, *SD* standard deviation

*Baseline adherence was evaluated during the 3 months prior to PCI

### Adherence rates for lipid-lowering medication

Tab. 2 presents lipid-lowering medication adherence after elective PCI for 2018, 2019, and 2020. Initial adherence (0–3 months post-PCI) was slightly above 70% each year and remained stable thereafter. Long-term adherence (10–12 months post-PCI) was slightly higher in 2020 (72.7%) compared to 2019 (71.5%) and 2018 (70.5%). Approximately 10% of patients were non-users of lipid-lowering medication in the first three months post-elective PCI, increasing to 13% during the year following PCI.

**Table 2:** Adherence to lipid-lowering medication during 1 year following elective percutaneous coronary intervention

			Period following PCI (months)			
*Year*	*Total elective PCI*		* 0–3*	* 4–6*	* 7–9*	*10–12*
*2018*	*n* *=* *16,686*	Adherent to LLM, %	72.3	71.6	71.2	70.5
		Not adherent to LLM, %	18.3	19.4	17.2	16.6
		LLM non-users, %	9.4	9.0	11.6	12.9
*2019*	*n* *=* *16,181*	Adherent to LLM, %	71.3	71.0	71.5	71.5
		Not adherent to LLM, %	19.1	19.3	16.4	15.6
		LLM non-users, %	9.6	9.7	12.1	12.9
*2020*	*n* *=* *13,607*	Adherent to LLM, %	73.2	72.8	72.9	72.7
		Not adherent to LLM, %	16.7	17.0	15.0	14.4
		LLM non-users, %	10.1	10.2	12.1	12.9

*LLM* lipid lowering medication, *PCI* percutaneous coronary intervention, *n* number

Adherent to LLM is defined as a medication possession rate of at least 80% in a certain period. Patients with a LLM prescription but with a medication possession rate of less than 80% are defined as not adherent. Patients without a LLM prescription in a certain period (medication possession rate of 0%) are defined as LLM non-users

Tab. 3 shows lipid-lowering medication adherence after acute PCI for 2018, 2019, and 2020. Patients’ adherence prior to acute PCI was lower compared to elective PCI. Initial adherence post-acute PCI was approximately 80%. Adherence rates declined during follow-up, with long-term rates lower than initial adherence rates (73.5% vs 80.3% in 2018, 74.9% vs 79.4% in 2019, and 76.3% vs 80.6% in 2020). Long-term adherence post-acute PCI was 3.0–3.6% higher than post-elective PCI. Long-term adherence post-acute PCI was slightly higher in 2020 (76.3%) compared to 2019 (74.9%) and 2018 (73.5%). In the first 3 months following acute PCI, less than 4% of patients were non-users of lipid-lowering medication, increasing to 9% during the year following PCI. Overall, these percentages are lower compared to elective PCI.

**Table 3:** Adherence to lipid-lowering medication during 1 year following acute percutaneous coronary intervention

			Period following PCI (months)			
*Year*	*Total acute PCI*		* 0–3*	* 4–6*	* 7–9*	*10–12*
*2018*	*n* *=* *16,322*	Adherent to LLM, %	80.3	77.0	75.3	73.5
		Not adherent to LLM, %	16.1	19.8	17.6	17.7
		LLM non-users, %	3.6	3.2	7.1	8.8
*2019*	*n* *=* *17,196*	Adherent to LLM, %	79.4	76.8	76.1	74.9
		Not adherent to LLM, %	16.9	19.7	16.4	16.2
		LLM non-users, %	3.7	3.5	7.5	8.9
*2020*	*n* *=* *17,184*	Adherent to LLM, %	80.6	78.3	77.9	76.3
		Not adherent to LLM, %	15.7	18.0	15.0	15.2
		LLM non-users, %	3.7	3.7	7.1	8.5

*LLM* lipid lowering medication, *PCI* percutaneous coronary intervention, *n* number

Adherent to LLM is defined as a medication possession rate of at least 80% in a certain period. Patients with a LLM prescription but with a medication possession rate of less than 80% are defined as not adherent. Patients without a LLM prescription in a certain period (medication possession rate of 0%) are defined as LLM non-users

### Adherence rates for lipid-lowering medication subtypes

**Fig. 2:**
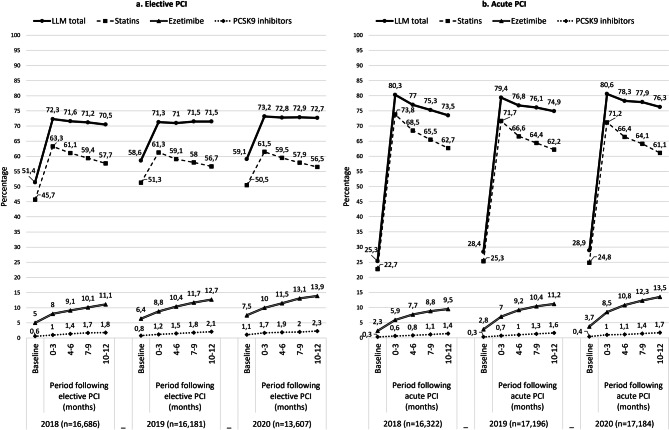
Adherence rates for lipid-lowering medication during 1 year following elective and acute percutaneous coronary intervention. (*LLM* lipid-lowering medication, defined as a medication possession rate of at least 80% in a certain period. Baseline adherence refers to adherence in the three months prior to PCI)

Figure 2 shows adherence rates for lipid-lowering medication subtypes in elective PCI patients, indicating an increase of 3.1–3.9% for ezetimibe and 0.6–0.9% for PCSK9 inhibitors, alongside a decrease of 4.6–5.6% for statins over the year following PCI. Adherence rates for ezetimibe and PCSK9 inhibitors in each period of 2020 were higher than in similar periods in 2019 and 2018. Similar patterns were observed post-acute PCI (Fig. 2), with adherence rates increasing by 3.6–5.0% for ezetimibe and 07–0.9% for PCSK9 inhibitors. However, the decrease in adherence rates for statins was higher (9.5–11.1%), resulting in an overall decline of 4.3%–6.8% in total lipid-lowering medication adherence.

### Adherence rate trends for statin subtypes

**Fig. 3:**
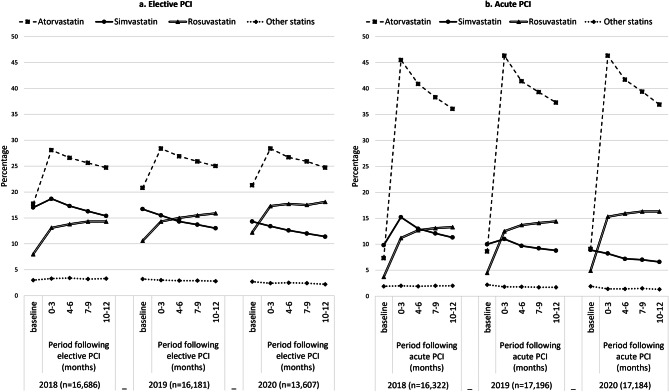
Adherence rates for different statins during 1 year following elective and acute percutaneous coronary intervention. (*LLM* lipid-lowering medication, defined as a medication possession rate of at least 80% in a certain period. Baseline adherence refers to adherence in the three months prior to PCI)

Adherence rate patterns for statin subtypes following elective and acute PCI are shown in Fig. 3. Post-acute PCI, atorvastatin usage was higher (36.1–46.3%) compared to elective PCI (24.7–28.4%). Over the year following both acute and elective PCI, rosuvastatin adherence increased by 0.8–2.1%, while simvastatin adherence decreased by 1.6–3.9%. Over the years, rosuvastatin adherence rates showed an increasing trend, whereas simvastatin adherence rates declined. Atorvastatin adherence rates remained relatively stable, while adherence rates for other statins slightly decreased over time (See Electronic Supplementary Material [ESM] TabS 1 and TabS 2).

### Adherence rates for lipid-lowering medication stratified by sex, age, recurrent PCI, and mortality

In secondary analyses, we stratified adherence rates for lipid-lowering medication by sex, age, and single/recurrent PCI. Adherence rates for males were 2.8–6.5% higher than for females across each period following elective and acute PCI (ESM Fig. S1). Among patients aged ≥ 80 years, adherence rates after both elective and acute PCI were about 7% lower compared to patients < 80 years. Still, long-term adherence rates for older patients were 64–66% post-elective PCI and 68–69% post-acute PCI (ESM Fig. S2). Adherence rates for patients with recurrent PCI within one year were about 2% higher compared to single PCI (ESMFig. S3). Adherence rates for patients who died before the end of the follow-up period were lower in comparison to the patients who survived the 1‑year follow-up period (ESM Fig S4.).

### Clinical practice variation

Long-term adherence to lipid-lowering medication after both elective and acute PCI for each of the 30 PCI centres is shown in the Electronic Supplementary Material Fig S5 and Fig S6. Post-elective PCI, adherence to lipid-lowering medication per centre varied between 57.9% to 76.2%, with an average long-term adherence of 71.5% across all centres. Post-acute PCI, adherence to lipid-lowering medication varied between centres from 68.5% to 80.7%, with an average long-term adherence of 74.9%. Adherence rates per region only slightly varied (ESM Fig S7 and S8).

## Discussion

This observational study using real-world nationwide data found that 71–81% of patients adhered to lipid-lowering medication during the year following PCI. For elective PCI patients, initial lipid-lowering medication adherence was 71–73%, remaining relatively stable throughout the year. Acute PCI patients showed higher initial adherence of 79–81%, declining to 74–76% over the year. Non-adherence rates were quite higher post-elective PCI (13% at 10–12 months) compared to post-acute PCI (9% at 10–12 months).

The higher initial adherence rate for acute PCI might be related to the mental impact of the acute setting. As a result, the motivation to take lipid-lowering medication for the prevention of a recurrent event is likely to be higher. In addition, acute PCIs are often followed by a hospital admission, while elective PCIs mostly do not require an overnight stay. This hospitalization may serve as a teachable moment that motivates patients to adopt positive cardiovascular health behaviour, including adherence to medications, in an effort to prevent further disease [19]. Also, as during an admission a patient is more exposed to different physicians, we can speculate that the chance to receive a prescription for lipid-lowering medication is probably higher.

Previous studies have reported similar lipid-lowering medication adherence rates. A 2012 meta-analysis reported a 76% adherence rate for statins as secondary preventive medication. Recent studies from Sweden and Norway on statin adherence after myocardial infarction showed 73% and 84% adherence rates, respectively [7, 8]. In our study, 14–20% of the patients had a lipid-lowering medication prescription but were nonadherent during the year following PCI. Being non-adherent and having an MP R < 80% could be explained by several factors. First, discontinuation of lipid-lowering medication, resulting in no new prescription. Second, extended intervals between dispenses due to various reasons, like forgetfulness or non-daily intake. Third, reduced medication frequency due to side effects. Unfortunately, this study could not identify specific reasons for non-adherence. Previous research has shown that patients not starting secondary preventive treatment shortly after acute myocardial infarction rarely start their treatment later, emphasizing the importance of immediate lipid-lowering medication initiation after PCI [7, 20]. Statin intolerance is the most significant cause of decreased adherence. Among statin side effects, muscle symptoms are the most commonly reported and a significant reason for discontinuation [21]. Other factors contributing to non-adherence include lack of knowledge, scepticism about statins’ necessity and effectiveness, insufficient information on side effects, infrequent lipid monitoring, and high-intensity statin prescriptions [22].

Throughout the year following PCI, we saw a decrease in statin use, but an increase in the use of ezetimibe and PCSK9 inhibitors. This is in line with previous studies reporting an increasing use of ezetimibe and PCSK9 inhibitors [23, 24]. Over time, familiarity with these lipid-lowering medication subtypes likely increased their prescriptions and use, especially when patients experienced statin side effects. As PSCK9 inhibitors will be off patent within a few years, it is expected that their use will further increase. All presented adherence rates, including ezetimibe and PCSK9 inhibitors, concern the dispensed medication. Although we can speculate that statins were replaced by ezetimibe and PCSK9 inhibitors, we can not draw such conclusions from this data.

This study showed adherence rates for various statin subtypes. Over the years, adherence to simvastatin decreased while adherence to rosuvastatin increased, indicating a shift in prescription rates and usage from simvastatin to rosuvastatin. This finding is in line with other literature [25]. Changes in the Dutch Cardiovascular Risk Management guideline in 2019, which removed specific statin preferences, might explain these trends [26]. Previously, simvastatin was recommended as it was cheaper, whereas rosuvastatin was a last resort due to its cost [27, 28]. With these guidelines, clinicians might have preferred the more potent rosuvastatin [29].

Comparable to our study, lower adherence rates for preventive medication after acute myocardial infarction were seen in females and elderly patients [10, 22]. The reasons underlying lower statin utilization in women remain poorly understood. Some studies suggest that a combination of females being offered statin therapy less frequently, while declining and discontinuing treatment more frequently, accounts for these sex differences [22, 30]. Regarding the lower adherence rates in the elderly, different reasons are mentioned in the literature. A systematic review found, for example, that polypharmacy and the presence of other noncardiac comorbidities has an adverse impact on statin adherence and continuation in elderly patients [31]. In addition, the ESC guideline advises to discontinue statins in frail persons.

### Strengths and limitations

Strengths of our study include the extensive coverage of the Vektis database, ensuring representativeness of lipid-lowering medication adherence in the Netherlands. Medication prescription data from pharmacies provides a more accurate usage picture compared to medical records. The results, including hospital-specific adherence rates, were discussed by the PCI registration committee of the NHR. Here, PCI centres with lower adherence rates can improve by adopting best practices from higher adherence centres. However, the study has limitations: dispensation does not guarantee medication intake, and data on the exact medication prescriptions were not available. The database also lacks individual patient data and information on LDL target achievement or side effects. Due to legal issues, the Vektis data used for this quality project can not be linked on a patient level with the clinical data as registered within the quality registries of the NHR. Besides, the data cannot distinguish reasons for non-adherence or medication switching patterns, determine how often patients switched statins, or track multiple medication use, which possibly explains the increased adherence to ezetimibe and PCSK9 inhibitors and decline for statins post-PCI.

## Conclusion

This observational study, using real-world nationwide data, found lipid-lowering medication adherence 1 year post-elective PCI ranged from 71–73% and post-acute PCI from 74–76%. Lower adherence rates were observed in women and elderly patients. The adherence rates of ezetimibe increased throughout the year following PCI to about 13%, and PCSK9 to about 2%, while statin use decreased.

## Supplementary Information

**FigS1.** Adherence rates for lipid-lowering medication during 1 year following elective and acute percutaneous coronary intervention, stratified by sex. Footnote: *LLM* = lipid-lowering medication, defined as a medication possession rate of at least 80% in a certain period. Baseline adherence refers to adherence in the three months prior to PCI.

**Fig S2**. Adherence rates for lipid-lowering medication during 1 year following elective and acute percutaneous coronary intervention, stratified by age groups. Footnote: *LLM* = lipid-lowering medication, defined as a medication possession rate of at least 80% in a certain period. Baseline adherence refers to adherence in the three months prior to PCI.

**Fig S3**. Adherence rates for lipid-lowering medication during 1 year following elective and acute percutaneous coronary intervention, stratified by single and recurrent PCI. Footnote: *LLM* = lipid-lowering medication, defined as a medication possession rate of at least 80% in a certain period. Baseline adherence refers to adherence in the three months prior to PCI.

**Fig S4**. Adherence rates for lipid-lowering medication during 1 year following elective and acute percutaneous coronary intervention, stratified by mortality. Footnote: *LLM* = lipid-lowering medication, defined as a medication possession rate of at least 80% in a certain period. Baseline adherence refers to adherence in the three months prior to PCI.

**Fig S5.** Long-term adherence for lipid-lowering medication after elective PCI—results per PCI centre. Footnote: *LLM* = lipid-lowering medication, defined as a medication possession rate of at least 80% in a certain period.

**Fig S6.** Long-term adherence for lipid-lowering medication after acute PCI—results per PCI centre. Footnote: *LLM* = lipid-lowering medication, defined as a medication possession rate of at least 80% in a certain period.

**Fig S7.** Adherence rates for lipid-lowering medication during 1 year following elective and acute percutaneous coronary intervention, stratified by region. Footnote: *LLM* = lipid-lowering medication, defined as a medication possession rate of at least 80% in a certain period. Baseline adherence refers to adherence in the three months prior to PCI.

**Fig S8.** Adherence rates for lipid-lowering medication during 1 year following elective and acute percutaneous coronary intervention, stratified by SES-region. Footnote: *LLM* = lipid-lowering medication, defined as a medication possession rate of at least 80% in a certain period. Baseline adherence refers to adherence in the three months prior to PCI.

**Fig S8.** Adherence rates for lipid-lowering medication during 1 year following elective and acute percutaneous coronary intervention, stratified by SES-region. Footnote: *LLM* = lipid-lowering medication, defined as a medication possession rate of at least 80% in a certain period. Baseline adherence refers to adherence in the three months prior to PCI.

## References

[1] Kerr AJ, Broad J, Wells S, Riddell T, Jackson R. Should the first priority in cardiovascular risk management be those with prior cardiovascular disease? Heart. 2009;95(2):125. 18381374 10.1136/hrt.2007.140905

[2] Visseren FLJ, Mach F, Smulders YM, et al. 2021 ESC Guidelines on cardiovascular disease prevention in clinical practice. Euro Heart J. 2021;42(34):3227–37. 10.1093/eurheartj/ehab484 34458905

[3] Cholesterol Treatment Trialists Collaborators. The effects of lowering LDL cholesterol with statin therapy in people at low risk of vascular disease: meta-analysis of individual data from 27 randomised trials. The Lancet. 2012;380(9841):581–90. 10.1016/S0140-6736(12)60367-5 PMC3437972 22607822

[4] Cholesterol Treatment Trialists’ Collaboration, Baigent C, Blackwell L, et al. Efficacy and safety of more intensive lowering of LDL cholesterol: a meta-analysis of data from 170,000 participants in 26 randomised trials. Lancet. 2010;376(9753):1670–81. 21067804 10.1016/S0140-6736(10)61350-5 PMC2988224

[5] Ference BA, et al. Low-density lipoproteins cause atherosclerotic cardiovascular disease. Evidence from genetic, epidemiologic, and clinical studies. A consensus statement from the European Atherosclerosis Society Consensus Panel. Eur Heart J. 2017;38(32):2459–72. 28444290 10.1093/eurheartj/ehx144 PMC5837225

[6] Gencer B, Marston NA, Im K, et al. Efficacy and safety of lowering LDL cholesterol in older patients: a systematic review and meta-analysis of randomised controlled trials. Lancet. 2020;396(10263):1637–43. 33186535 10.1016/S0140-6736(20)32332-1 PMC8015314

[7] Halvorsen S, et al. Initiation of and long-term adherence to secondary preventive drugs after acute myocardial infarction. BMC Cardiovasc Disord. 2016;16:115. 27246583 10.1186/s12872-016-0283-6 PMC4886431

[8] Jernberg T, et al. Cardiovascular risk in post-myocardial infarction patients: nationwide real world data demonstrate the importance of a long-term perspective. Eurn Heart Journal. 2015;36(19):1163–70. 10.1093/eurheartj/ehu505 25586123

[9] Naderi SH, Bestwick JP, Wald DS. Adherence to Drugs That Prevent Cardiovascular Disease: Meta-analysis on 376,162 Patients. Am J of Med. 2012;125(9):882–887.e1. 22748400 10.1016/j.amjmed.2011.12.013

[10] Eindhoven DC, et al. Age and gender differences in medical adherence after myocardial infarction: Women do not receive optimal treatment – The Netherlands claims database. Eur J Prev Cardiol. 2020;25(2):181–9. 10.1177/2047487317744363 29164916

[11] Eindhoven DC, van Staveren LN, van Erkelens JA, et al. Nationwide claims data validated for quality assessments in acute myocardial infarction in the Netherlands. Neth Heart J. 2018;26(1):13–20. 29119544 10.1007/s12471-017-1055-3 PMC5758448

[12] Ray KK, Aguiar C, Arca M, et al. Use of combination therapy is associated with improved LDL cholesterol management: 1‑year follow-up results from the European observational SANTORINI study. Eur J Prev Cardiol. 2024;31(15):1792–803. 38861400 10.1093/eurjpc/zwae199

[13] Ray KK, Haq I, Bilitou A, et al. Treatment gaps in the implementation of LDL cholesterol control among high- and very high-risk patients in Europe between 2020 and 2021: the multinational observational SANTORINI study. Lancet Reg Health Eur. 2023;29:100624. 37090089 10.1016/j.lanepe.2023.100624 PMC10119631

[14] Ray KK, Molemans B, Schoonen WM, et al. EU-Wide Cross-Sectional Observational Study of Lipid-Modifying Therapy Use in Secondary and Primary Care: the DA VINCI study. Eur J Prev Cardiol. 2021;28(11):1279–89. 33580789 10.1093/eurjpc/zwaa047

[15] Timmermans MJC, Houterman S, Daeter ED, et al. Using real-world data to monitor and improve quality of care in coronary artery disease: results from the Netherlands Heart Registration. Neth Heart J. 2022;30(12):546–56. 35389133 10.1007/s12471-022-01672-0 PMC8988537

[16] De Staat van Volksgezondheid en Zorg. Number of people without health insurance - trends throughout the years [Available from: https://www.staatvenz.nl/kerncijfers/onverzekerden-zorgverzekering].

[17] Anghel LA, Farcas AM, Oprean RN. An overview of the common methods used to measure treatment adherence. Med Pharm Rep. 2019;92(2):117–22. 31086837 10.15386/mpr-1201 PMC6510353

[18] Ministry of Health, Welfare and Sport. Socio-economic status per region [Available from: https://www.vzinfo.nl/sociaaleconomische-status/regionaal/algemeen].

[19] Fonarow GC. In-hospital initiation of statins: taking advantage of the ’teachable moment’. Cleve Clin J Med. 2003;70(6):502–6. 12828221 10.3949/ccjm.70.6.502

[20] Gislason GH, Rasmussen JN, Abildstrøm SZ, et al. Long-term compliance with beta-blockers, angiotensin-converting enzyme inhibitors, and statins after acute myocardial infarction. Eur Heart J. 2006;27(10):1153–8. 16399775 10.1093/eurheartj/ehi705

[21] Stroes ES, Thompson PD, Corsini A, et al. Statin-associated muscle symptoms: impact on statin therapy-European Atherosclerosis Society Consensus Panel Statement on Assessment, Aetiology and Management. Eur Heart J. 2015;36(17):1012–22. 25694464 10.1093/eurheartj/ehv043 PMC4416140

[22] Ingersgaard MV, et al. Reasons for Nonadherence to Statins - A Systematic Review of Reviews. Patient Prefer Adherence. 2020;14:675–91. 32308373 10.2147/PPA.S245365 PMC7135196

[23] Katzmann JL, et al. Trends in Ezetimibe Prescriptions as Monotherapy or Fixed-Dose Combination in Germany 2012-2021. Front Cardiovasc Med. 2022;9:912785. 35770230 10.3389/fcvm.2022.912785 PMC9234160

[24] Talic S, Marquina Hernandez C, Ofori-Asenso R, et al. Trends in the Utilization of Lipid-Lowering Medications in Australia: An Analysis of National Pharmacy Claims Data. Current Problems in Cardiology. 2022;47(7)100880. 34108083 10.1016/j.cpcardiol.2021.100880

[25] Aarnio E, et al. Socioeconomic Inequalities in Statin Adherence Under Universal Coverage. Circulation: Cardiovascular Quality and Outcomes. 2016;9(6):704–13. 27756795 10.1161/CIRCOUTCOMES.116.002728

[26] Dutch 2019 Cardiovascular Risk Management guidelines (In Dutch: NHG standaard Cardiovasculair risicomanagement). Dutch 2019 Cardiovascular Risk Management guidelines (In Dutch: NHG standaard Cardiovasculair risicomanagement). 2019. https://richtlijnen.nhg.org/standaarden/cardiovasculair-risicomanagement.

[27] Food and Drug Administration, Department of Health & Human Services. ANDA approval 079167. 2016.

[28] Astra Zeneca. AstraZeneca settles litigation over CRESTOR patent - Press Release (updated 25 March 2013). 2013. https://www.astrazeneca.com/media-centre/press-releases/2013/astrazeneca-crestor-patent-litigation-settled-25032013.html!.

[29] Jones PH, Davidson MH, Stein EA, et al. Comparison of the efficacy and safety of rosuvastatin versus atorvastatin, simvastatin, and pravastatin across doses . Am J Cardiol. 2003;92(2):152–60. 12860216 10.1016/s0002-9149(03)00530-7

[30] Nanna MG, Wang TY, Xiang Q, et al. Sex Differences in the Use of Statins in Community Practice. Circ Cardiovasc Qual Outcomes. 2019;12(8)e005562. 31416347 10.1161/CIRCOUTCOMES.118.005562 PMC6903404

[31] Ofori-Asenso R, Jakhu A, Curtis AJ, et al. A Systematic Review and Meta-analysis of the Factors Associated With Nonadherence and Discontinuation of Statins Among People Aged >65 Years. J Gerontol A Biol Sci Med Sci. 2018;73(6):798–805. 29360935 10.1093/gerona/glx256

