# Supplementary figures and images for: Medication adherence to lipid-lowering agents after percutaneous coronary intervention: nationwide real-world data in the Netherlands
Source: Neth Heart J. 2026 Mar 2;34(4):143–52. doi: 10.1007/s12471-026-02028-8 (PMC13009448; doi:10.1007/s12471-026-02028-8)

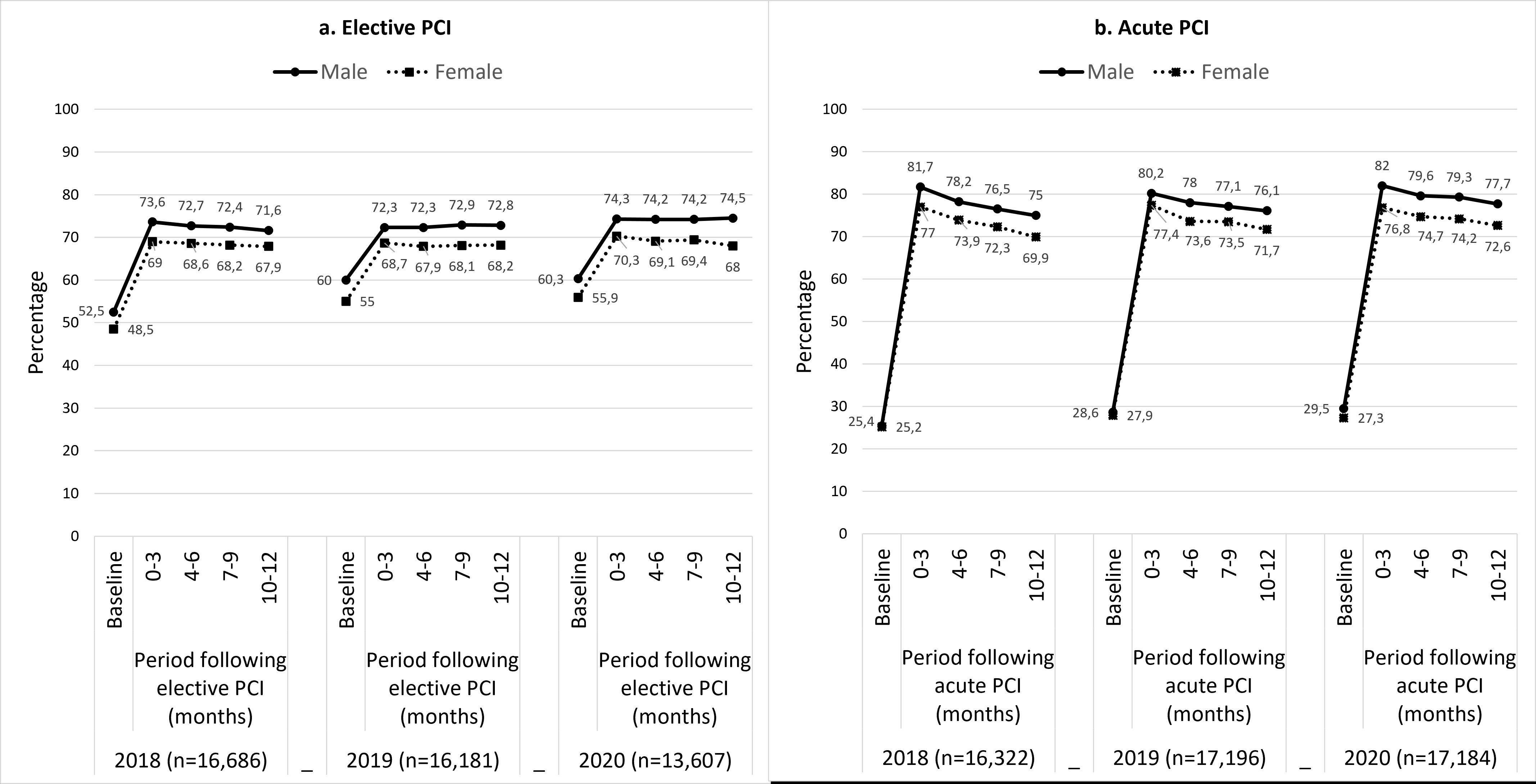

Supplement: Supplementary file 1 — FigS1. Adherence rates for lipid-lowering medication during 1 year following elective and acute percutaneous coronary intervention, stratified by sex. Footnote: LLM = lipid-lowering medication, defined as a medication possession rate of at least 80% in a certain period. Baseline adherence refers to adherence in the three months prior to PCI. [file 12471_2026_2028_MOESM1_ESM.jpg]

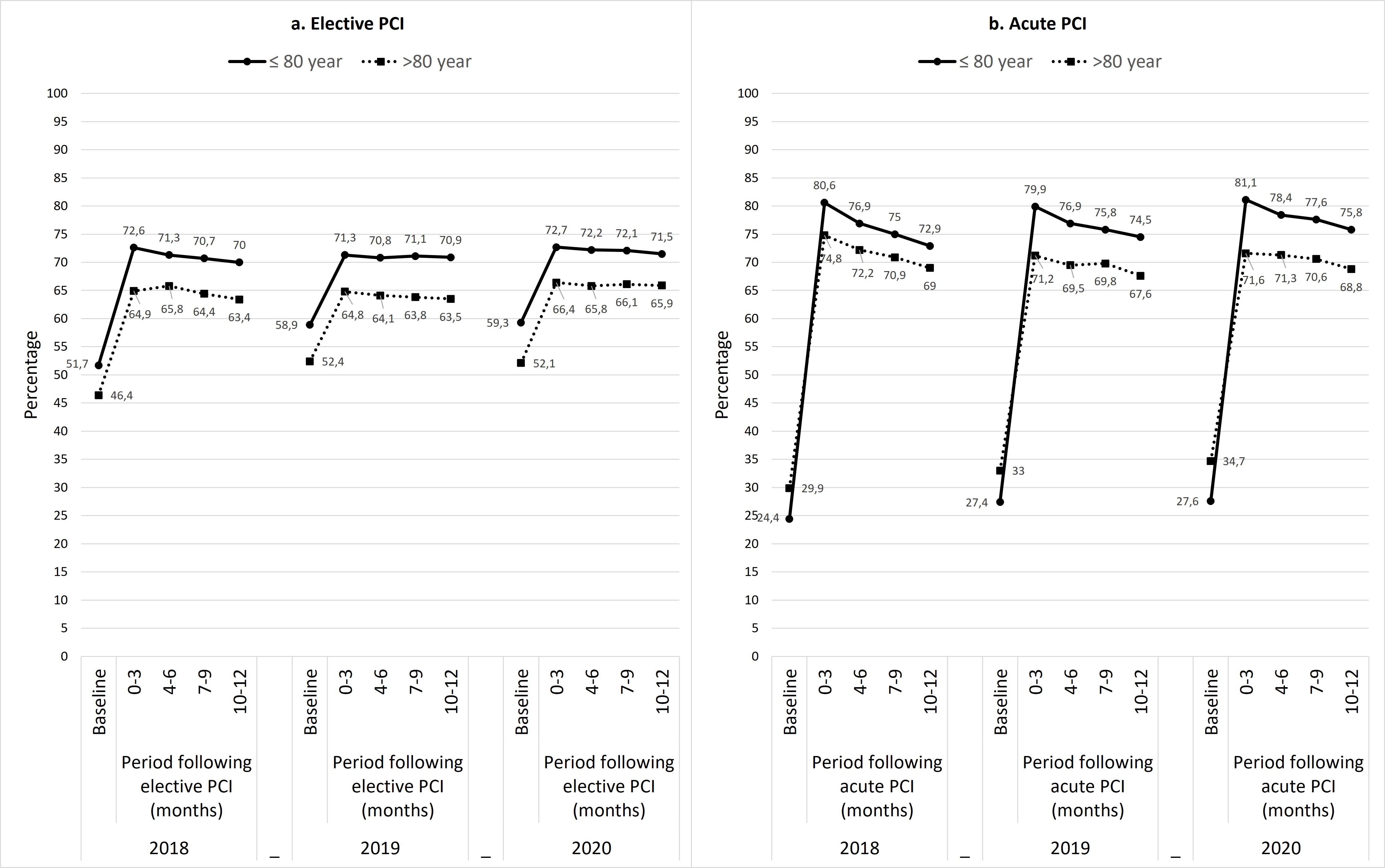

Supplement: Supplementary file 2 — Fig S2. Adherence rates for lipid-lowering medication during 1 year following elective and acute percutaneous coronary intervention, stratified by age groups. Footnote: LLM = lipid-lowering medication, defined as a medication possession rate of at least 80% in a certain period. Baseline adherence refers to adherence in the three months prior to PCI. [file 12471_2026_2028_MOESM2_ESM.jpg]

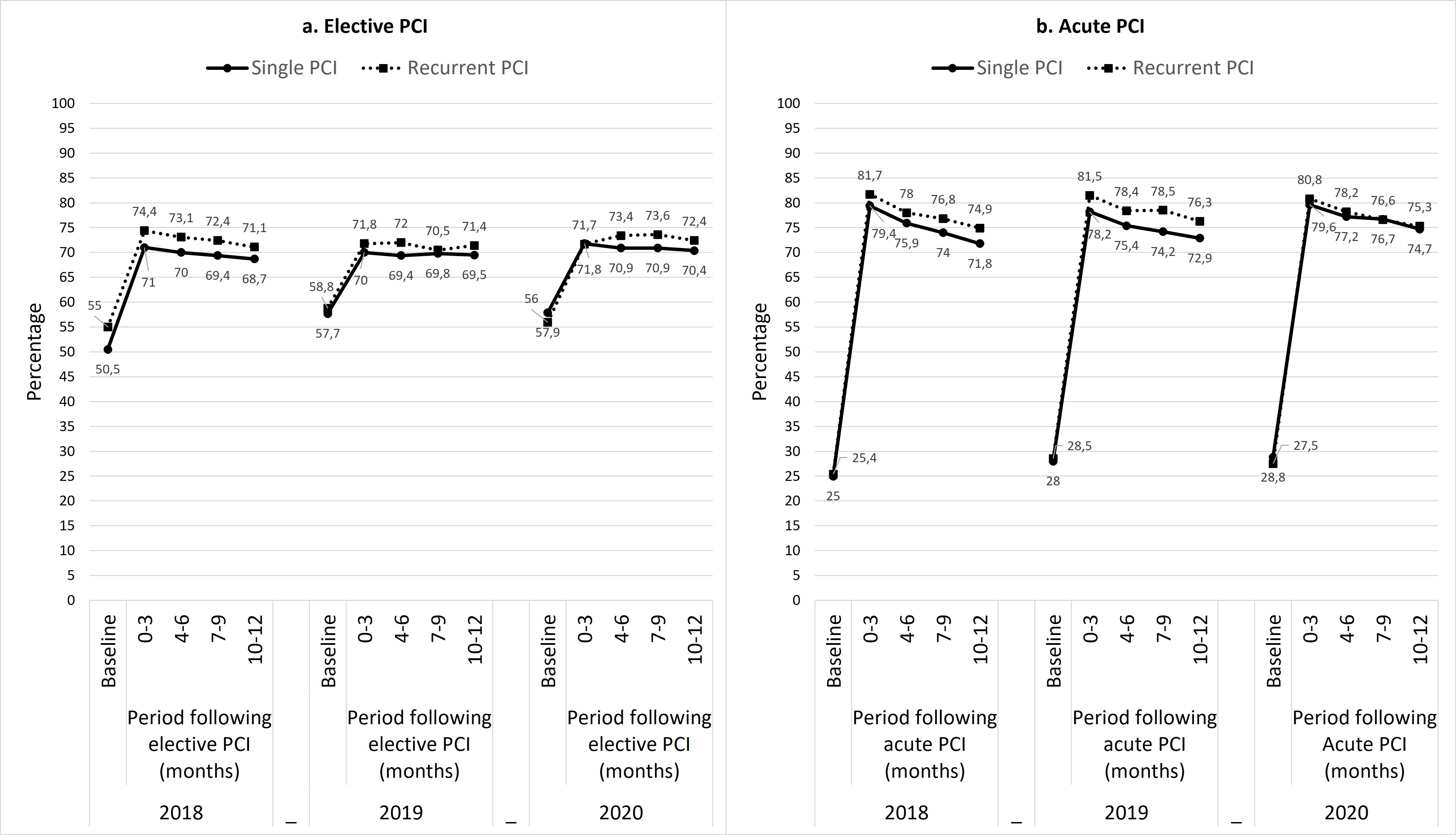

Supplement: Supplementary file 3 — Fig S3. Adherence rates for lipid-lowering medication during 1 year following elective and acute percutaneous coronary intervention, stratified by single and recurrent PCI. Footnote: LLM = lipid-lowering medication, defined as a medication possession rate of at least 80% in a certain period. Baseline adherence refers to adherence in the three months prior to PCI. [file 12471_2026_2028_MOESM3_ESM.jpg]

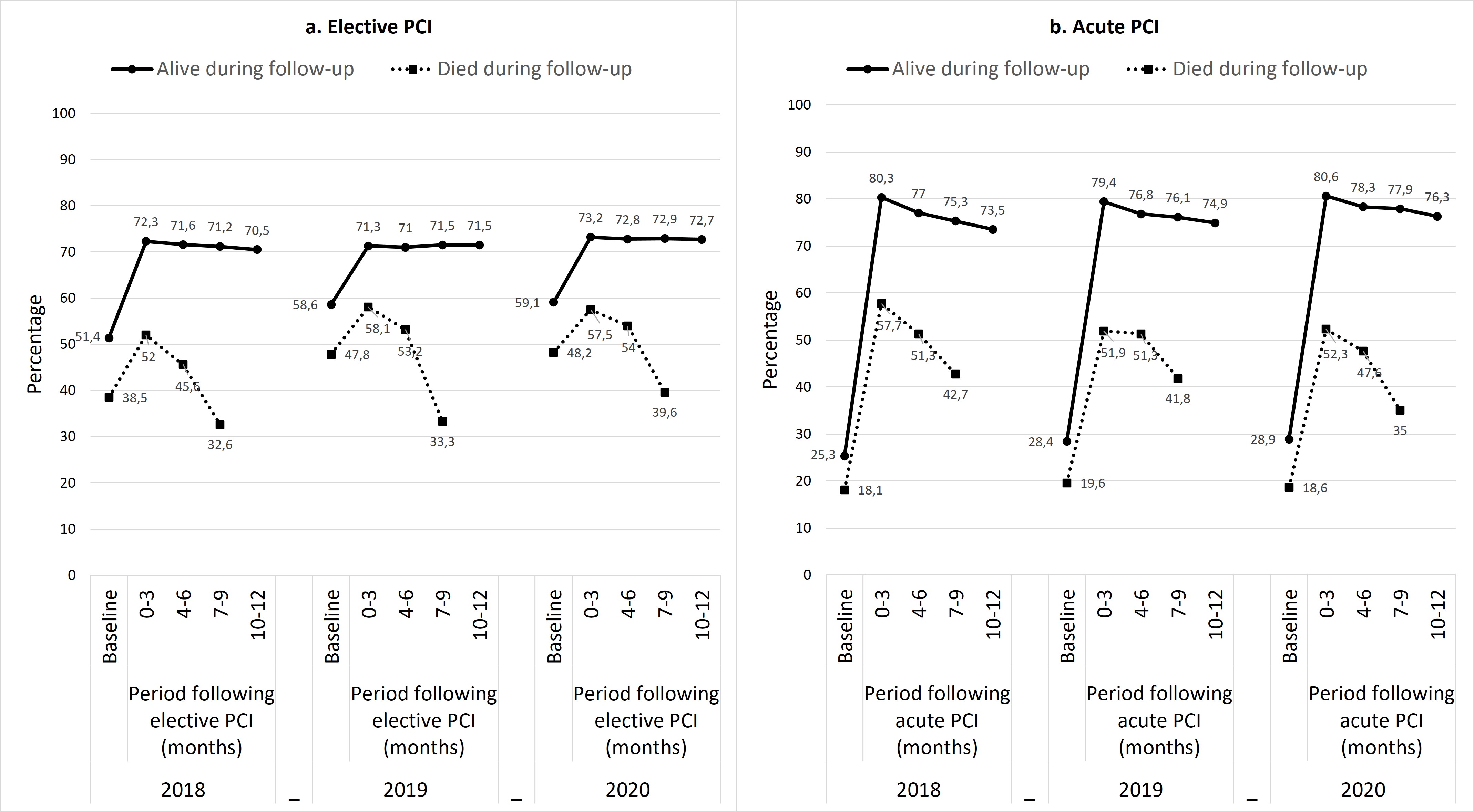

Supplement: Supplementary file 4 — Fig S4. Adherence rates for lipid-lowering medication during 1 year following elective and acute percutaneous coronary intervention, stratified by mortality. Footnote: LLM = lipid-lowering medication, defined as a medication possession rate of at least 80% in a certain period. Baseline adherence refers to adherence in the three months prior to PCI. [file 12471_2026_2028_MOESM4_ESM.jpg]

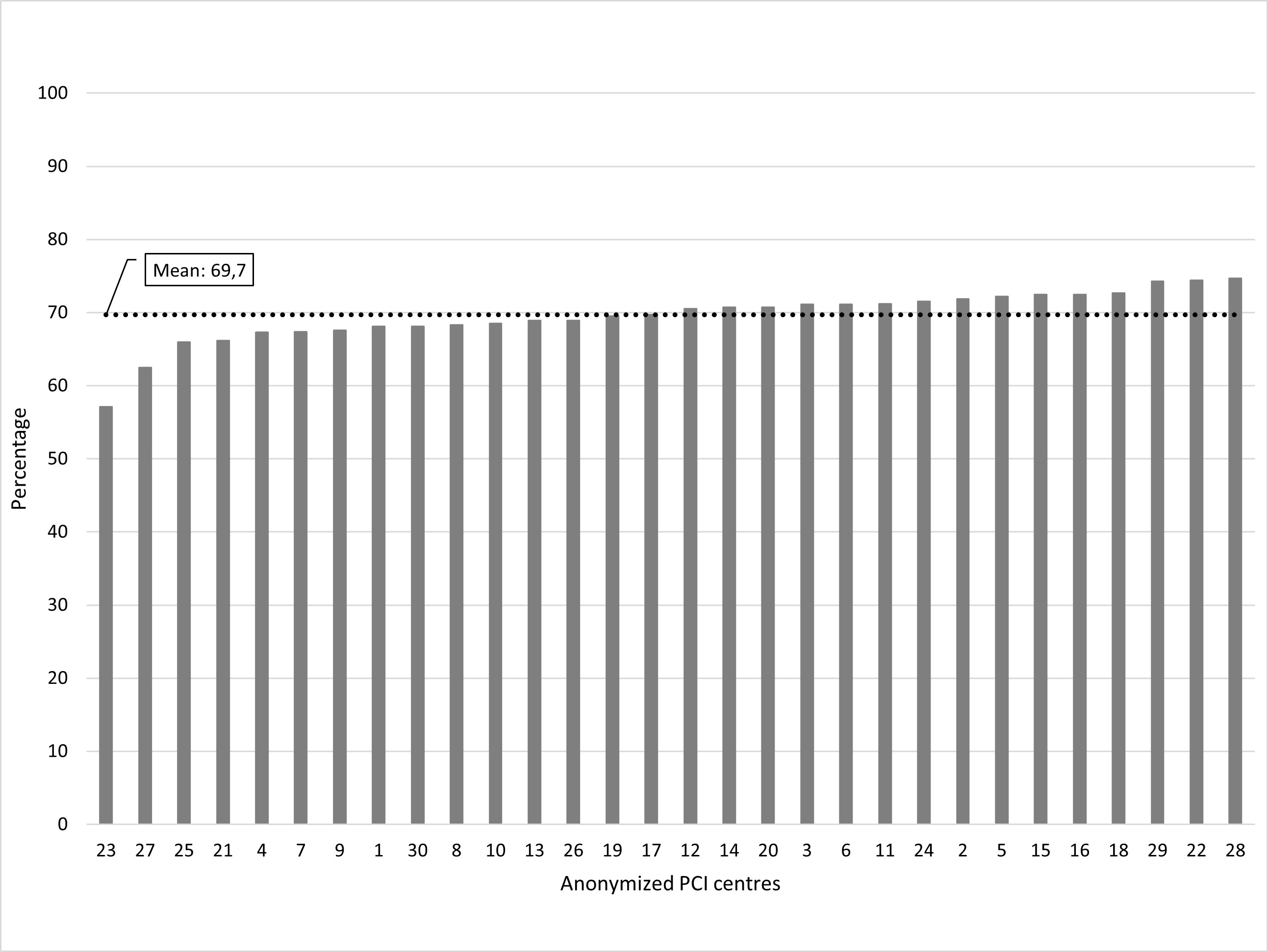

Supplement: Supplementary file 5 — Fig S5. Long-term adherence for lipid-lowering medication after elective PCI—results per PCI centre. Footnote: LLM = lipid-lowering medication, defined as a medication possession rate of at least 80% in a certain period. [file 12471_2026_2028_MOESM5_ESM.jpg]

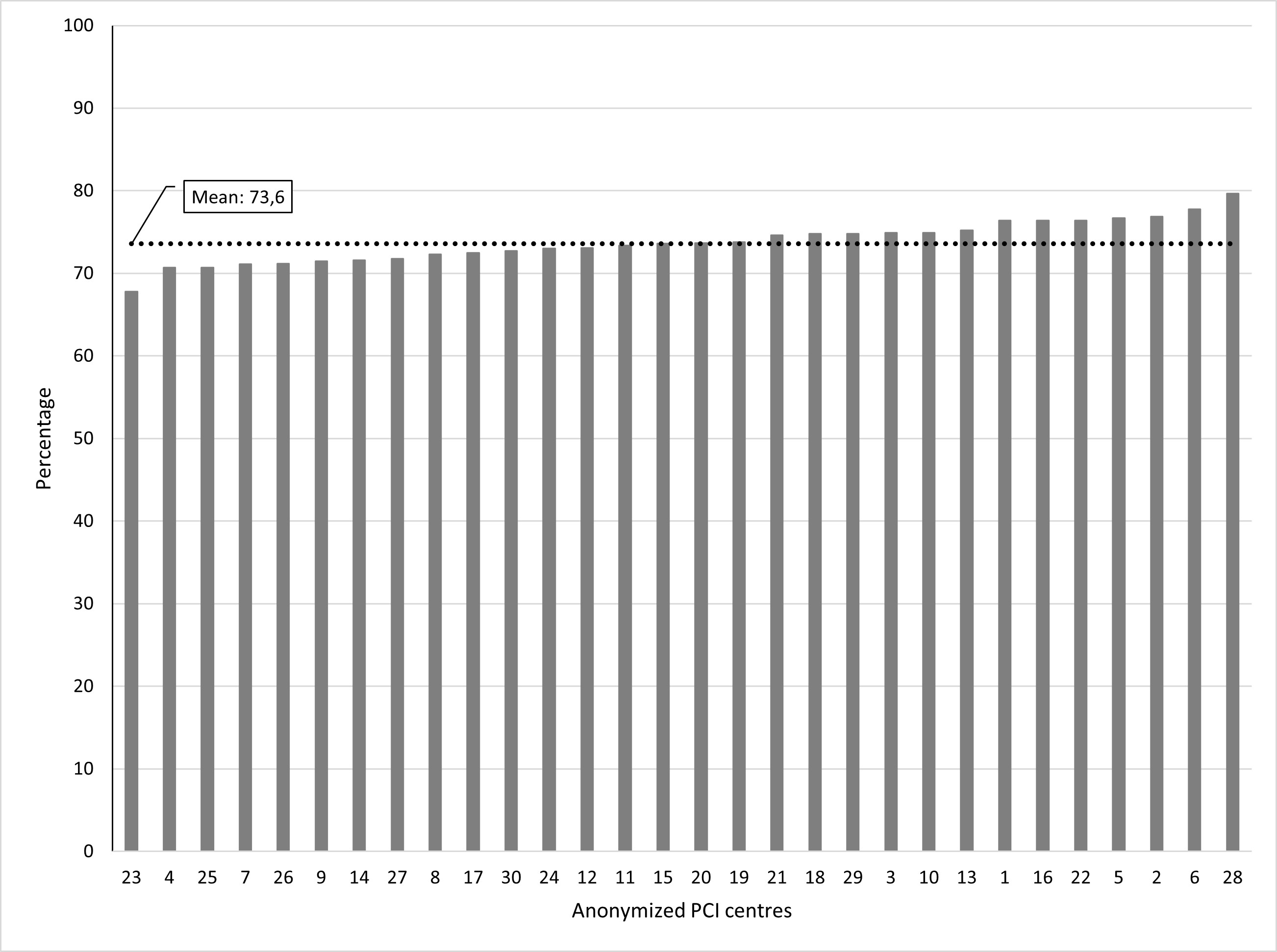

Supplement: Supplementary file 6 — Fig S6. Long-term adherence for lipid-lowering medication after acute PCI—results per PCI centre. Footnote: LLM = lipid-lowering medication, defined as a medication possession rate of at least 80% in a certain period. [file 12471_2026_2028_MOESM6_ESM.jpg]

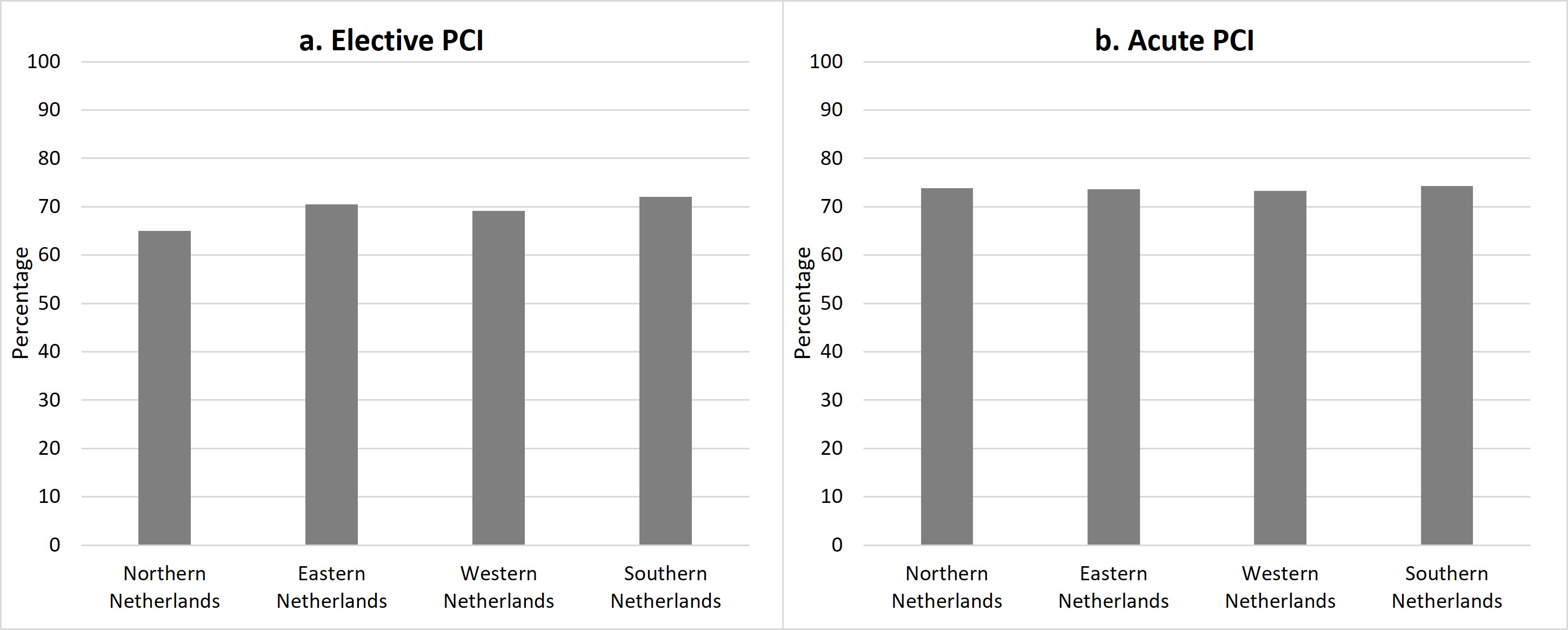

Supplement: Supplementary file 7 — Fig S7. Adherence rates for lipid-lowering medication during 1 year following elective and acute percutaneous coronary intervention, stratified by region. Footnote: LLM = lipid-lowering medication, defined as a medication possession rate of at least 80% in a certain period. Baseline adherence refers to adherence in the three months prior to PCI. [file 12471_2026_2028_MOESM7_ESM.jpg]

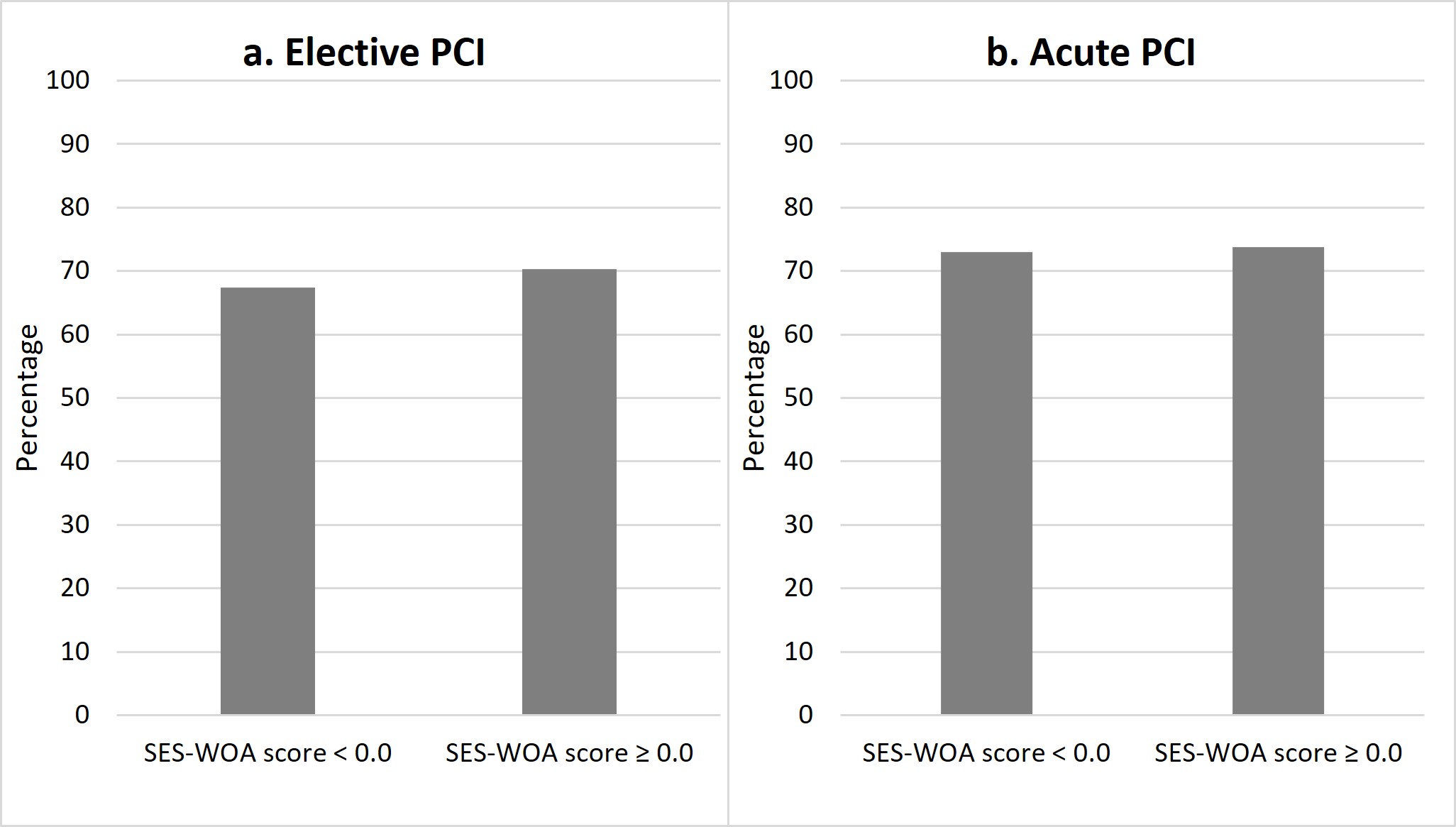

Supplement: Supplementary file 8 — Fig S8. Adherence rates for lipid-lowering medication during 1 year following elective and acute percutaneous coronary intervention, stratified by SES-region. Footnote: LLM = lipid-lowering medication, defined as a medication possession rate of at least 80% in a certain period. Baseline adherence refers to adherence in the three months prior to PCI. [file 12471_2026_2028_MOESM8_ESM.jpg]
