# Supplementary material for: Medication adherence to lipid-lowering agents after percutaneous coronary intervention: nationwide real-world data in the Netherlands
Source: Neth Heart J. 2026 Mar 2;34(4):143–52. doi: 10.1007/s12471-026-02028-8 (PMC13009448; doi:10.1007/s12471-026-02028-8)
Supplement: Supplementary file 9 — Fig S8. Adherence rates for lipid-lowering medication during 1 year following elective and acute percutaneous coronary intervention, stratified by SES-region. Footnote: LLM = lipid-lowering medication, defined as a medication possession rate of at least 80% in a certain period. Baseline adherence refers to adherence in the three months prior to PCI. [file 12471_2026_2028_MOESM9_ESM.docx]

| **Name** | **Centre** |
| --- | --- |
| Dr. J.M. Cheng | Albert Schweitzer ziekenhuis |
| Dr. M. Meuwissen | Amphia |
| Dr. M. Grundeken | Amsterdam UMC |
| Dr. K. Teeuwen | Catharina Ziekenhuis |
| Dr. H. Al Hashimi | CWZ |
| Dr. S. Hubbers | Elisabeth-TweeSteden Ziekenhuis |
| Dr. R. Diletti | Erasmus MC |
| Dhr. B.J. Sorgdrager | Haaglanden Medisch Centrum |
| Dhr. C.E. Schotborgh | HagaZiekenhuis |
| Dr. T. Meijers | Isala |
| Dr. J. Polad | Jeroen Bosch Ziekenhuis |
| Dr. R. Scherptong | Leids Universitair Medisch Centrum |
| Dr. E. Bakker | Maasstad Ziekenhuis |
| Prof. dr. A.J.W. van ‘t Hof | Maastricht UMC+ |
| Dr. F. Spano | Meander Medisch Centrum |
| Dhr. J. Brouwer | Medisch Centrum Leeuwarden |
| Dhr. K.G. van Houwelingen | Medisch Spectrum Twente |
| Dr. M. Ewing | Noordwest Ziekenhuisgroep |
| Dr. G. Amoroso | OLVG |
| Dhr. C. Camaro | Radboudumc |
| Dr. P.W. Danse | Rijnstate |
| Dr. K. Sjauw | St. Antonius Ziekenhuis |
| Dr. R. van Bommel | Tergooi |
| Dhr. W.T. Ruifrok | Treant Zorggroep |
| Dr. A.O. Kraaijeveld | UMC Utrecht |
| Dr. E. Lipsic | Universitair Medisch Centrum Groningen |
| Dr. L. Hoebers | VieCuri Medisch Centrum |
| Dhr. R. Erdem | ZorgSaam Zorggroep Zeeuws-Vlaanderen |
| Dr. L. Ruiters | Zuyderland Medisch Centrum |

**Appendix 1. Members of the PCI Registration Committee**
